# Migration and health: exploring healthy ageing of immigrants in European societies

**DOI:** 10.1017/S1463423623000014

**Published:** 2023-02-03

**Authors:** Frode F. Jacobsen, Stinne Glasdam, Limke M. Schopman, Morten Sodemann, Maria E.T.C. van den Muijsenbergh, Gudmund Ågotnes

**Affiliations:** 1 Centre for Care Research, Western Norway, Western Norway University of Applied Services, P.O.Box 7030, N-5020 Bergen, Norway; VID Specialized University, Ulriksdal 10, N-5009, Bergen, Norway; 2 Integrative Health Research, Department of Health Sciences, Faculty of Medicine, Lund University, Margaretavägen 1 B, S- 222 41, Lund, Sweden; 3 Self-Employed, Pastoor Havinkstraat 37, 7561 AS, Deurningen, the Netherlands; 4 The Migrant Health Clinic, Odense University Hospital, Sdr. Boulevard 29, 5000 Odense, Denmark; 5 GP and Professor of Health Disparities and Personcentred Integrated Primary Care, Radboud University Medical Centre, Nijmegen, the Netherlands; Pharos, National Centre of Expertise on Health Disparities, Utrecht, the Netherlands; 6 Department of Welfare and Participation, Western Norway University of Applied Sciences, Inndalsveien 28, 5063 Bergen, Norway

**Keywords:** healthy ageing, immigrants, older persons, primary health care

## Abstract

**Aim:**

The aim is to identify important factors for immigrants’ health and well-being and for their use (or non-use) of primary health care (PHC) and other non-specialised services, and for possible ways that PHC can support healthy ageing of immigrants.

**Background:**

Older persons are an increasing share of the immigrant population in the global north, frequently in contact with various forms of health services, (PHC services most of all. Consequently, PHC services are in a particularly unique position to support healthy ageing of immigrants.

**Methods:**

The position paper builds on five international, multi-professional and cross-disciplinary small group discussions as well as an international workshop early summer. During the discussions and the workshop, topics were arrived at as to factors related to the health situation of older immigrants, their needs, and health-seeking behaviour, and to how PHC professionals could support healthy ageing in immigrants. Those main topics in turn guided search for relevant research literature and informed the selection of the main research questions of this paper.

**Findings:**

Several factors, in addition to culture and cultural differences, are important to for PHC professionals and decision-makers to take into consideration in encounters with older immigrants. The socio-economic position of the older immigrant and close relatives, inter-generational relationships within the immigrant communities, country-specific factors in the host country like health care expenditure, and communication skills in health professionals are all examples of factors playing an important role regarding the health and health-seeking behaviour of older immigrants.

## Introduction

The number of immigrants across the global north has increased substantially during the latter decades. This has sparked a growing interest in the general topics of migration and integration, as well as questions relating to health, well-being, and functional status. A growing proportion of immigrants are older persons, frequently in contact with various forms of health services, primary health care (PHC) services most of all. Consequently, PHC services are in a particularly unique position to support healthy ageing of immigrants. Hence, the primary questions raised in this position paper are as follows:

1. What are the main opportunities and obstacles for healthy ageing of immigrants in a European context?
2. How can PHC support healthy ageing of immigrants?

Ageing and immigration are both complex multidimensional processes shaped by a range of factors at different levels over the life course of an individual, including cultural dimensions from both country of origin and residence. It will be argued that even though culture and cultural understanding is important, several un-related factors also influence how immigrants in general and older immigrants in particular fare as to health and well-being, and these factors will hence be address these in this paper. When culture is at stake and central in the current paper, it is important to make explicit the cultural position of the authors. All the authors are carriers of Western cultures, where they are all non-immigrants in the various European countries where they reside.

## Aim

Through exploring the questions below, the current paper aims to identify important factors for immigrants’ health and well-being and for their use (or non-use) of PHC and other non-specialised services, including long-term care services.

The questions are as follows:

1. What characterises the health situation of the older immigrant populations?
2. What characterises older immigrants’ health-seeking behaviour?
3. Which factors influence older immigrants’ health-seeking behaviour?
4. How could competence in cross-cultural consultations support healthy ageing among immigrants?

For supporting healthy ageing among immigrants, examples of challenges to health and to seeking proper professional help to be discussed are as follows: (1) socio-economic conditions; (2) social isolation due to language, communication difficulties, and cultural barriers; (3) lack of professional interpreters and professional guidelines for cross-cultural communication not being implemented in practice; (4) stressful relationships of help between the generations; and (5) conflict between values and habits of the original home country and new hybrid values developed in the younger generations. Finally, the paper aims at identifying promising measures for supporting healthy ageing of older immigrants.

## Background

There has been a dramatic increase in international migration over the past few decades, and it is continuously rising (International Organisation for Migration IOM. (2020) World Migration Report 2020. Geneva, Switzerland: International Organisation for Migration, 498 pp. https://publications.iom.int/system/files/pdf/wmr_2020.pdf Jiang *et al*., 2019; Hossin, 2020). In 2020, the total population living in countries other than their place of birth was estimated to 281 million, compared to 153 million in 1990, equivalent to 3.6% of the global population (International Organization for Migration, 2020). In 2020, Europe is the largest international migration destination with 87 million immigrants (International Organization for Migration, 2020). Culture is important for understandings of well-being, health, illness, and the logics of health care systems. Conceptions of the body, health, and healing are all “spun in a web of meaning” where culture is part and parcel of both experiences of health and illness and of health-seeking strategies of people, regardless of cultural and social backgrounds (Jacobsen, 1998). Neither lay conceptions nor scientific and professional conceptions of health and illness are devoid of culture (Stein, 1990; Sontag, 2002). Cultural differences in such concepts do pose challenges for communication in clinical settings (Debesay, 2012; Schouten *et al*., 2020), as do cultural differences in when old age sets in, what does old age imply and what is successful ageing (Wray, 2003; Torres, 2006; Bowling, 2009; Zubair & Norris, 2015; Frackowiak *et al.*, 2020). As an example of the latter, for older immigrants, successful ageing may alternatively imply mastering or delaying signs of ageing, surrendering to, and accepting changes associated with the ageing body, or living in harmony with one’s ageing body (Torres, 2006). Dominant taken-for-granted views in the majority population in the host country may be a pronounced barrier for cross-cultural communications in clinical encounters and beyond. What is at stake for immigrants in clinical encounters, however, is about much more than culture, as will be discussed in the following.

Access to health care is inequitably distributed. Socio-economic background, and related to it, health literacy, seem to play a major role for access, with immigrants being one of the groups associated with low socio-economic status and low health literacy. For several countries in Europe and beyond, PHC is the first point of entry for the general population. Strengthening PHC is a priority of several European countries, and for good reasons, since PHC plays a central role in recognising needs as well in the coordination of health and care services. Recent research points towards a strong inverse relationship between the strength of a country’s PHC system and social inequity of unmet needs (Detollenaere *et al*., 2017). Furthermore, research shows immigrant populations are vulnerable to serious health disparities, with many immigrants experiencing significantly worse health outcomes than non-immigrants. Consequently, these health risks demand effective health communication. However, while the need for effective communication about general health and well-being is particularly acute, it is also tremendously complicated to communicate effectively with this population of immigrants (e.g. Kreps & Sparks, 2008). Immigrants are commonly shaped by social, cultural, economic and political factors in their country of origin while experiencing the new social-cultural and political environment in the country of destination (Castañeda *et al*., 2015). Still, older immigrants comprise a heterogeneous population. They derive from a broad list of countries, varying much in terms of social, social-economic and political characteristics. While some of them are first-generation immigrants, like labour immigrants of the 1960s and early 1970s, other have been born in the host country. The older persons who have themselves experienced migration have migrated for various reasons, from fleeing war and persecution to work immigration and forced displacement because of environmental disasters. Moreover, while some have immigrated during old age, others have been children, youths or adults at the time of migration. However, older immigrants and their children may have intergenerational struggles based on them being reunified in recipient country without consent or prior knowledge, and because of differences in values and lifestyles, as the older immigrant adults often maintain their inherited cultures while the second/third immigrant generations are more likely to adapt to the host cultures (Liu & Reeves, 2015). The differences in values and lifestyles are moreover a source of psychological and social stress in relations of care across the generations.

The socio-economic status of immigrants may also vary considerably before and after immigration (Gustafsson *et al*., 2021). In general, older immigrants tend to have less income and capital than the majority population, which again may cause chronic stress and related morbidity. Age at migration appears to be a strong predictor for labour market integration, and time until a first entry into the labour market increases rapidly with age at migration, a situation which has severe implications for their socio-economic position (Mac Innes, 2022). Their legal status in the host country is of primary importance, where illegal, undocumented older immigrants make up a highly vulnerable population. The fact that they make much less use of health care services than the majority population adds to their vulnerability (van den Muijsenbergh *et al*., 2014).

## Methods

The position paper builds on five international, multi-professional, and cross-disciplinary small group discussion within the PRIMORE network, as well as an international workshop early summer 2022 at the Western University of Applied Sciences in Bergen, Norway, where immigrant health was one of the major topics. During the discussions and the workshop, topics were arrived at as to factors related to the health situation of older immigrants, their needs, and health-seeking behaviour, and to how PHC professionals could support healthy ageing in immigrants. Those main topics in turn guided search for relevant research literature, where both literature reviews and presentations of original research were sought. Moreover, they informed the selection of the main research questions of this paper and the related topics.

### Findings

#### Some health and socio-economic situation of older immigrants

The lower the income of older immigrants and their families, the more they will suffer for unmet needs, as evidenced by data from the European Union Statistics on Income and Living Conditions database (Detollenaere *et al*., 2017). Albeit immigrants represent a broad range of socio-economic backgrounds, a majority tend to be in the lower echelon of income and hence particularly prone to experience inequalities in health (Detollenaere *et al.*, 2017). Study of income data from Denmark and Sweden, in example, reported much higher poverty rates among the immigrants than among non-immigrants (Gustafsson *et al.*, 2021).

Also in old age, income inequalities may be pronounced and be related to inequalities in health. A study including 16 Western European EU countries found substantial income inequality among retirees among immigrants from non-EU countries. Income gaps appeared to be smaller in countries where the pension system is more distributive (Heisig *et al.*, 2018).

In general, immigrants appear to have a worse self-reported health than the majority population. Increasing evidence suggests that migration is an independent social determinant of illness and health (Denktas *et al.*, 2009; Lanari *et al.*, 2015; Sodeman, 2022). As an example, a recent Norwegian study found older immigrants to be worse off than non-immigrant Norwegians in a range of aspects of health and functioning (Qureshi *et al*., 2022).

A so-called healthy immigrant effect (HIE) has been researched for several years, frequently explained by a combination of a positive self-selection of healthy immigrants and selective process through screening and discrimination in the host countries (Constant *et al.*, 2018; Markides & Rote, 2019). To what extent HIE exists is disputed nowadays. However, immigrants’ health status tends to decrease with time spent in the host country (Sodeman, 2022). Older immigrants often suffer from a high level of comorbidity and disability, a finding that may be explained by intersection of ethnicity, social class, and gender (Markides & Rote, 2019; Holman & Walker, 2021).

Presence of intergenerational differences, clearly having a cultural dimension, adds to the general stress experienced by older immigrants, not the least in relation to sexuality, where opposing views can be a source of conflicts (Sodeman, 2022). Younger generations have access to information channels not controlled by the older generation, whose members may hence experience that their traditional authority is questioned. Being torn between so-called traditional and modern worldviews and habits are common human experiences. Still, immigrants often find themselves in more vulnerable situation in this regard, feeling pressured by norms of sexuality of the host society, for example, as to norms of sexuality and gender roles (Sodeman, 2022).

In old age, values dominating in the country of origin tend to surface, including language use. This is not the least of importance in the palliative phase when death approaches. This is one of several important changes in older immigrants that appear to be universal rather than culture-specific (Sodeman, 2022), increasing the need for both language and cultural translation.

#### What characterises the health-seeking behaviour of older immigrants?

Regarding health-seeking behaviour, several factors shape how and to what extent immigrants use health and care services. A study from the Netherlands (2016) showed less than half of the undocumented immigrants indicated to visit the general practitioner for health care issues (Watjer *et al.*, 2016). Immigrants tend to make less use of some of the specific health care options than the general population, amongst other, public health facilities, screening, and preventive programmes, and homecare provisions (van den Muijsenbergh *et al.*, 2014; Schouten *et al.*, 2020), partly because of communication difficulties and partly because of cultural differences (Hovde *et al.*, 2008a; 2008b). In some areas also the effectiveness of services, such the effectiveness of treatment, seem lower for immigrants than non-immigrants (Huber *et al*., 2008). In Norway, researchers found a lower share of immigrants (74.2%) than non-immigrants (83.3%) reported having visited a doctor the previous year (Qureshi *et al*., 2022). Some immigrants are deeply rooted in collectivist values, resulting in changing information and concealing the truth to protect the family’s collective interest (Li *et al*., 2021; Fu & Glasdam, 2022). However, western health care systems commonly are based on the individual according to personal needs, whereas the older immigrant may represent a more collective attitude (Priebe *et al.*, 2011), which may cause cultural and ethical challenges in encounters between some immigrants and Western health care systems. To tackle those possible barriers, Watjer *et al*. (2016) suggest increasing transparency and communication on the health care situation of immigrants towards the public and, even so, improving knowledge of both undocumented immigrants and PHC professionals on the health care rights.

There are indications that also older immigrants utilise health services less than the native-born population. A Dutch study reported a systematic lower health services utilisation of older immigrants in the four major immigrant groups in the Netherlands (Denktas *et al*., 2009).

In a Danish study, older immigrants were identified as less likely than ethnic Danes to use municipal long-term care services, with the biggest difference between ethnic Danes and immigrants from non-Western countries who lived few years in Denmark, a difference that decreases with increasing time in the host country. Lack of language skills and of knowledge of the health care system may be two possible factors behind those findings (Hansen, 2014).

### Factors influencing health-seeking behaviour

Country-specific factors like level of expenditures on health and physician density are associated with higher immigrant usage of physician services (Solé-Auró *et al.*, 2012). Characteristics of the host countries are, in other words, significant in how older immigrants’ fare. The host countries vary substantially in terms of legal systems and socio-economic support as well as to organisation of health care, both at the provider and system level. Hence, the fate of immigrants seems to vary with host country. As one example, a study of older people born in Yugoslavia or Turkey who had immigrated to Denmark the same period and with similar characteristics found that they were more likely to be in poverty than the ones who had immigrated to Sweden (Gustafsson *et al*., 2021).

Intergenerational support is important throughout the individual life course and a major mechanism of cultural continuity (Bordone & de Valk, 2016). Although intergenerational support in the event of illness varies much among immigrants regarding country of origin and to the host country (Bordone & de Valk, 2016), a more collectivistic approach as to persons with health or other needs seems more frequent in many immigrant communities, where a common attitude is that the whole family network needs help (Debesay, 2012; Johannessen *et al*., 2013; Sodeman, 2022). Peer education can be useful for information dissemination. A study in the Netherlands pointed out that most information is communicated from person to person within one’s social network. Using social media was considered an important means of communication to reach a large group of immigrants (Watjer *et al.*, 2016). However, there is also a need to acknowledge a generation gap in use of social media and, in general, a tendency towards a digital exclusion of older people (Gallistl *et al*., 2020). Still, whether communication is digital or not, having a family perspective is most important for involved health professionals (Sodeman, 2022). However, as already pointed out, generational differences can add stress to relations of care within families.

Communication skills in health professionals are of uttermost importance for older immigrants. PHC professionals often struggle with communication barriers in meeting their needs (Watjer *et al*., 2016). Examples are numerous as to potential possibilities for misunderstandings and miscommunication. Some immigrants may experience, for example, issues related to sexuality. However, they may choose to present their problems as depression, as a less sensitive label for their problems. Immigrants may have had experienced humiliation when sharing sensitive information with health professionals without being properly understood. Others may have toothache, for example, but since fixing this problem is conceived to be expensive, they present the problems as migraine, knowing that it is possible and not too expensive to get medicine for the pain of migraine. Yet others may have multiple health complaints but have the conception that one should only present the doctor with one problem, and hence the problem is framed as pain in the whole body. Some immigrants may experience that a particular diagnosis, for example, related to a type of functional disorder and that they do not agree with still stick to them, while they do not have the courage or energy to attempt to correct it (Sodeman, 2022). The result of miscommunication is likely to be solutions that do not function for the person (Sodeman, 2022), including a greater tendency towards accessing emergency services (van den Muijsenbergh *et al.,* 2014).

There is a pronounced need for professional interpreters in health care services, especially for older immigrants. Even though engagement of professional interpreters is not without its challenges, for example, related to political and cultural differences the interpreter and the older person, various studies have shown that use of professional interpreters can support and strengthen communication, use of health care services, compliance, and treatment results and reduce hospital stays and medication errors (Debesay, 2012; Sodeman, 2022). Still, such interpreters appear to be rarely used in health care services (van den Muijsenbergh *et al.*, 2014; Sodeman, 2022). Close family members may experience burnout from being employed as interpreters, sometimes even since childhood (Debesay, 2012; Sodeman, 2022). As an alternative, cooperation between grassroots organisations, migrant organisations and churches in activities targeting access to health care and knowledge exchange can be extended to partly overcome this gap (Watjer *et al*., 2016).

Older immigrants are, as highlighted, a highly diverse category. It is significant for immigrants’ understandings and uses of various health care services at what stage in their life they immigrated. It is also important under which circumstances they migrated as to their health and care needs. Traumatic experiences of war, torture, and violent death of close relatives and friends tend to stay with them for them for the rest of their lives (Kim *et al*., 2019), as do traumatic experiences related to the transit to final host country (Peterie, 2018). Additionally, as already mentioned, their legal status makes a big difference as to needs and for their health-seeking behaviour (van den Muijsenbergh *et al.*, 2014; Schouten *et al*., 2020).

### The need for competence in cross-cultural consultations to improve healthy ageing

There is a documented need for increased competence in cross-cultural consultations in primary care. However, despite the existence of a broad range of professional guidelines, these are rarely implemented in practice. There is a dearth of knowledge on what conditions need to be in place for their implementation and why (van den Muijsenbergh *et al.*, 2014; Schouten *et al*., 2020). The question could be asked whether the guidelines are to extensive, cumbersome and labour-intensive to be put into daily clinical use, or whether there are other factors related to health care practices that pose obstacles.

Understanding older immigrants require the questioning of widespread stereotypes. While stereotypes in general can be necessary for relating to the world and everyday life for health professionals and others, stereotypes easily get in the way when trying to relate to the unique persons with unique combination of needs and in unique circumstances (Debasay, 2012; Sodeman, 2022). Guidelines directed to specific cultural or national groups, although something can always be learned from them, may still be a hindrance rather than a help in this endeavour. Even though gaining knowledge about cultural and national background is necessary, providing room for the story of the older immigrant is of paramount importance (van den Muijsenbergh *et al.*, 2014; Sodeman, 2022).

### Overarching recommendations

At a system level, policymakers should focus on reducing income inequality to tackle inequity in access in the society per se and strengthen primary care and long-term care services, by increasing accessibility and developing its workforce. There is a need for both PHC professionals, policymakers and researchers to identify and understand barriers to appropriate use of health care services by older immigrants. There is furthermore a need for understanding factors that facilitates or hinders development of quality in care for older immigrants, probably for all persons with an immigrant background.

At an organisation and facility level, strengthening the use of professional interpreters is an important goal to better communication, service use and health outcomes, not the least in services for older immigrants. In addition, avoiding extensive use of family members and relatives as interpreters spare those informal interpreters of stressful and sometimes harmful situations.

At the level of both individual health services and professionals, cultivating a professional openness among health professional colleagues, with an exchange of ideas that includes the individual person, may be a way forward, where who the person is, what are the main vulnerabilities, and what is important for the person, are issues at the fore (Sodeman, 2022). Inter-cultural skills need to be developed. As part of this, the ability to deeply listen to the persons with an open mind and to be able to assess and learned from their stories is of uttermost importance. Culture and cultural differences are important to keep in mind. Still, there are a host of other important factors to consider that cannot be reduced to culture, like socio-economic status, reasons for immigration, experiences prior to and during immigration, legal status as immigrant as well as universal factors related to age, generations, and other characteristics. Taking the general life situation of each individual person into account is of primary importance.
